# *Helicobacter pylori* Infection Acts as an Independent Risk Factor for Intracranial Atherosclerosis in Women Less Than 60 Years Old

**DOI:** 10.3389/fcvm.2021.819315

**Published:** 2022-01-11

**Authors:** Yinjie Guo, Canxia Xu, Linfang Zhang, Zhiheng Chen, Xiujuan Xia

**Affiliations:** ^1^Department of Gastroenterology, The Third Xiangya Hospital of Central South University, Changsha, China; ^2^Department of Ophthalmology, The Second Xiangya Hospital of Central South University, Changsha, China; ^3^Department of Health Management, The Third Xiangya Hospital of Central South University, Changsha, China

**Keywords:** *Helicobacter pylori*, intracranial atherosclerosis, gender difference, age difference, endothelial dysfunction

## Abstract

**Background:** Studies show inconsistent results regarding the relationship between *Helicobacter pylori* (*H. pylori*) infection and stroke. The present study assessed a potential association between *H. pylori* infection and an important risk factor for stroke, intracranial atherosclerosis.

**Methods:** In total, 15,798 subjects with transcranial Doppler (TCD) and 13C-urea breath test (13C-UBT) were enrolled from March 2012 to March 2017. Intracranial atherosclerosis was further measured using intracranial carotid artery calcification (ICAC) on past or recent head CT, and 14,084 subjects were ultimately included in the study. Baseline demographics, atherosclerosis risk factors, and laboratory results were investigated. Since endothelial dysfunction is critical to the development of atherosclerosis, the role of *H. pylori* in migration, tube formation, and proliferation of human brain microvascular endothelial cells (HBMECs) was assessed *in vitro*.

**Results:** The intracranial atherosclerosis group had a higher proportion of women and a greater rate of *H. pylori* infection than those without intracranial atherosclerosis. *H. pylori* infection was significantly more common in women with intracranial atherosclerosis than males. In addition, the incidence of intracranial atherosclerosis was significantly higher in women with *H. pylori* infection than uninfected women (53.8 vs. 46.4%, *p* < 0.001). In an adjusted model, *H. pylori* was shown to be an independent risk factor for intracranial atherosclerosis in women ≤ 60 years of age [odds ratio (OR) = 2.261, 95% CI = 1.839–2.780, *p* < 0.001]. Serum exosomes from patients with *H. pylori* infection had significantly reduced brain endothelial cell migration, tube formation, and proliferation *in vitro*.

**Conclusion:** *Helicobacter pylori* infection may be an important independent risk factor for intracranial atherosclerosis in women ≤ 60 years of age.

## Introduction

*Helicobacter pylori* (*H. pylori*), a microaerophilic bacterium that colonizes the human gastric epithelium, is the causative agent for one of the most common bacterial infections worldwide (1). The prevalence of *H. pylori* ranges from 30 to 50% in developed countries to ~80% in developing countries, especially those in Asia, as a result of differences in environmental factors, individual lifestyle, and population-based characteristics (2–4). *H. pylori* is well recognized as the primary pathogen involved in peptic ulcer, chronic gastritis, and gastric cancer (5) and has been more recently associated with many non-gastrointestinal diseases, such as cardiovascular, neurological, hematological, metabolic, and skin disorders (6, 7).

Intracranial atherosclerosis is defined by wall thickening, non-stenosing plaques, or luminal narrowing of the intracranial arteries that can lead to transient or permanent cerebral ischemic events and is considered the major risk factor for stroke (8, 9). Age, arterial hypertension, type 2 diabetes mellitus (T2DM), and metabolic syndrome are the conventional risk factors for atherosclerosis (10, 11). However, studies indicate that 30–50% of patients lack these risk factors, suggesting that other factors influence pathogenesis (12). In addition, because atherosclerosis is a chronic inflammatory disease, ongoing chronic infection may impact disease development (13). Several studies have shown an association between *H. pylori* infection and stroke (14–17). A systematic review with the meta-analysis by Doheim et al. associated the presence of anti-*H. pylori* IgG with an increased risk of stroke [odds ratio [OR] (95% CI) = 1.43 (1.25–1.46)](15), and a retrospective study reported that patients with *H. pylori* infection had a higher incidence of ischemic stroke than uninfected patients (16). However, a few studies have found no association between *H. pylori* infection and stroke (18–20). In addition, studies reported controversial results on the association between *H. pylori* and other neurological pathologies, such as neurodegeneration (21–23). Metabolic syndrome is well known as the conventional risk factor for atherosclerosis and studies also have shown an association between *H. pylori* infection and metabolic syndrome (24, 25).

The mechanism of atherosclerosis pathogenesis is complex and has not been fully defined; however, endothelial dysfunction is believed to play a critical role (26). During infection, *H. pylori* invades and colonizes the gastric mucosal epithelial layer but it remains unknown how *H. pylori* induces endothelial dysfunction and promotes the development of atherosclerosis *in vivo*. Exosomes are found in many bodily fluids and play an essential role in cell-to-cell communication by transporting various bioactive constituents such as lipids, proteins, mRNA, and microRNAs (27, 28). Recent studies have shown that exosomes play a key role in transferring toxic proteins associated with neurodegenerative diseases (29). *H. pylori* virulence factor cytotoxin-associated gene A (CagA) is found in serum-derived exosomes isolated from patients with CagA^+^ *H. pylori* infection (30). The present study was designed to explore that *H. pylori* infection impair brain endothelial function through exosomes.

To date, the relationship between *H. pylori* infection and stroke remains controversial and the association between *H. pylori* infection and intracranial atherosclerosis, especially among young and asymptomatic patients, or those with mild symptoms, in the Chinese population, has not been well investigated. The present study assessed if *H. pylori* infection is associated with an increased risk of intracranial atherosclerosis.

## Materials and Methods

### Subjects

**Figure 1:**
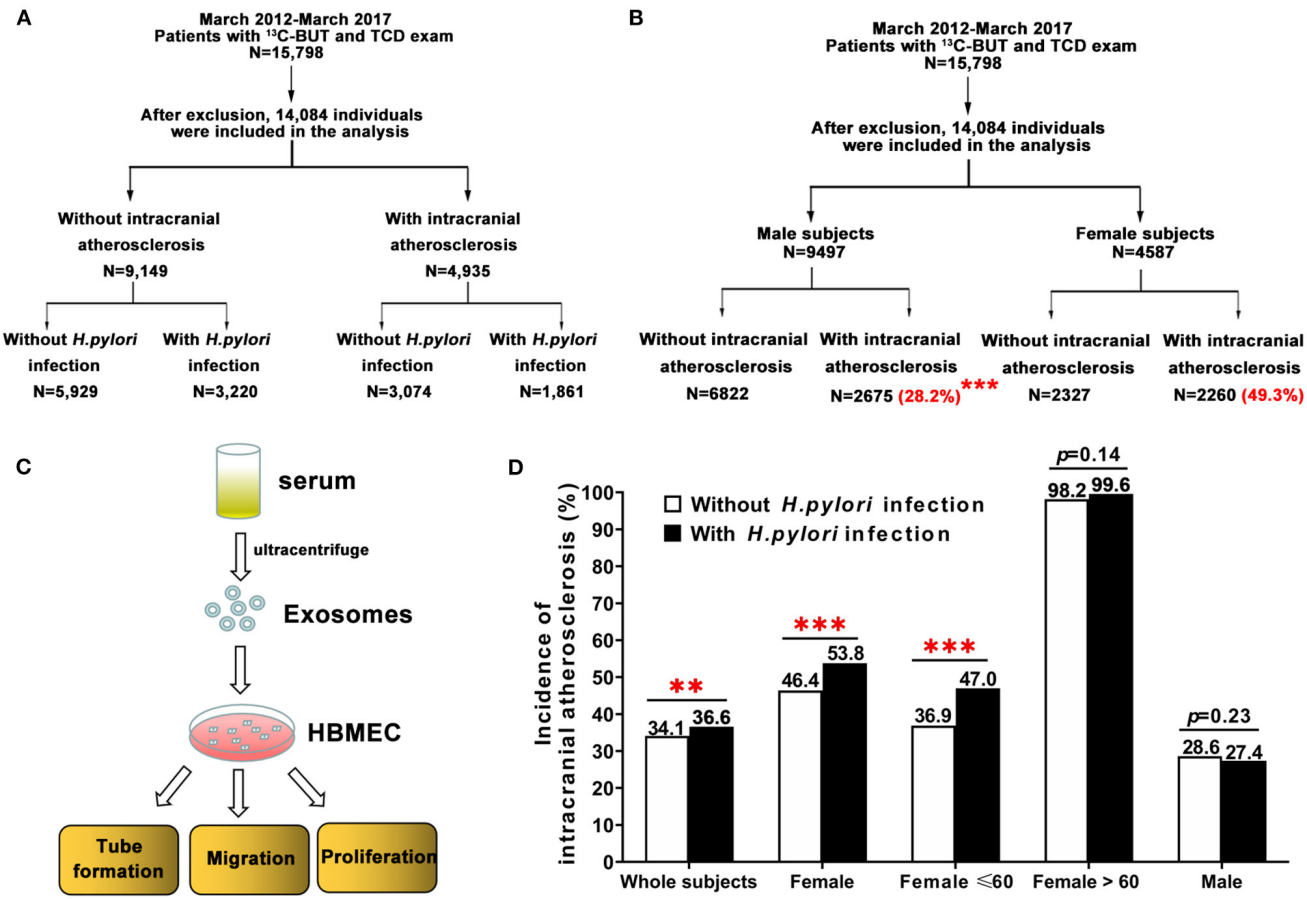
Study design and the incidence of intracranial atherosclerosis among individuals with and without *H. pylori* infection, stratified by age. **(A,B)** Subjects were screened and divided into different groups based on the study protocol. **(B)** Female subjects had a significantly higher prevalence of intracranial atherosclerosis than men (49.3 vs. 28.2%, ^***^p < 0.001). **(C)** To test the role of *H. pylori* on endothelial cell function, serum exosomes obtained from human subjects by ultracentrifugation were added to HBMEC to assess migration, tube formation, and proliferation. **(D)** There was a significant difference in the incidence of intracranial atherosclerosis between subjects with and without *H. pylori* infection (36.6 vs. 34.1%, ^**^p < 0.01). *H. pylori*-positive women had a significantly higher incidence of intracranial atherosclerosis than *H. pylori*-negative women (53.8 vs. 46.4%, ^***^p < 0.001). Further analysis showed age differences in *H. pylori*-infected women and incidence of intracranial atherosclerosis. However, there was no significant difference in intracranial atherosclerosis incidence between male subjects with and without *H. pylori* infection. HBMEC, human brain microvascular endothelial cells.

Subjects who received a transcranial Doppler (TCD) and 13C-urea breath test (13C-UBT) during their annual physical examination at the Third Xiangya Hospital of Central South University in Changsha, Hunan, China, from March 2012 to March 2017 were enrolled in this study. Individuals with a history of *H. pylori* eradication, use of any H2-receptor blockers, proton pump inhibitors or antibiotics 3 months prior to the test, age <20 or >80 years, malignancy, thyroid disease, asthma or chronic obstructive pulmonary disease (COPD), hematological disorder or abnormal liver function, or abnormal TCD results who do not have a past head CT result and refused a head CT, were excluded. Subjects were screened and divided into different groups according to the study protocol (Figures 1A,B). Written informed consent was obtained from all subjects prior to participation. The study was conducted according to the principles of the Declaration of Helsinki and was approved by the Clinical Research Ethics Committee of the Third Xiangya Hospital of Central South University, Changsha, China (Approval Number of Ethics Committee: 2019-S241).

### Assessment of Intracranial Atherosclerosis

Intracranial carotid artery calcification (ICAC) measured by head CT is shown to be a reliable marker for intracranial atherosclerosis (8, 31). Subjects with abnormal results on TCD (EME TC8080; Nicolet, Madison, WI, USA) without a past head CT result underwent non-enhanced head CT scanning for artery calcification. TCD examination was performed by an experienced neuroradiologist using a 2 MHz frequency ultrasound probe. Subjects were placed in the supine position after 10 min of rest. Four main acoustic windows, (1) transtemporal, (2) transorbital, (3) submandibular, and (4) suboccipital and blood flow, at various depths within each major branch of the circle of Willis were assessed. Head CT (Siemens, Erlanger, Germany) scans were performed by an experienced neuroradiologist. Bone window CT images (slice thickness 5 mm) through the skull base were used to identify the presence or absence of carotid arterial calcifications in each cavernous carotid (ICAC) artery (32). The presence of intracranial atherosclerosis was assessed by an independent expert reader, who was blinded to all clinical laboratory findings and other imaging data.

### Detection of *H. pylori* Infection

After fasting for at least 6 h, subjects received a two-stage 13C-UBT (H20174047, HaiDeRun, Beijing, China) to detect the presence of *H. pylori* infection. 13C infrared spectrometry was used to analyze exhaled breath samples from each patient. The receiver-operating characteristic curves were used to define the cut-off delta-over-baseline (DOB) values. DOB ≥ 4 (33) was considered positive and DOB <4 was considered negative.

### Other Baseline Measurements

Physical and laboratory test data were collected as described (34). Body mass index (BMI) equivalent to weight was divided by square of height (kg/m^2^). After overnight fasting, blood samples from the antecubital vein were obtained from each patient to measure glucose and serum lipids using a Hitachi 7170S autoanalyzer (Hitachi, Tokyo, Japan). Diabetes was diagnosed among patients with a fasting plasma glucose concentration >6.11 mmol/L or those taking anti-diabetic medicine. Resting blood pressures were obtained with an electronic sphygmomanometer after patients rested for 15 min and three readings from each patient were averaged. Arterial hypertension was diagnosed among patients with a systolic blood pressure (SBP) >130 mmHg, a diastolic blood pressure (DBP) >80 mmHg, or those taking anti-hypertensive medicine.

### Cell Culture

Human brain microvascular endothelial cells (HBMECs, ScienCell Research Laboratories, Carlsbad, CA, USA) were cultured in an endothelial cell medium (#1001, ScienCell Research Laboratories, Carlsbad, CA, USA) in a controlled humidified incubator with 5% CO_2_. To assess the role of *H. pylori* on brain endothelial cell function, serum exosomes (100 μg/ml) were isolated from subjects with CagA^+^ *H. pylori* infection or healthy controls, cultured with HBMEC, and collected to measure cell proliferation, migration, and tube formation (Figure 1C).

### Exosome Preparation

Serum samples were collected from five patients with CagA^+^ *H. pylori* infection or five healthy control subjects were combined to isolate an adequate number of exosomes, as previously described (35). In brief, exosomes were isolated by successive centrifugation at 4°C at increasing speeds of 2,000 *g* for 30 min and 12,000 *g* for 45 min and ultracentrifuged two times at 110,000 *g* for 2 h. Exosome pellets were re-suspended in a small volume of phosphate-buffered saline (PBS) for further analysis. A transmission electron microscope (TEM, TECNAI G2 Spirit; FEI, Hillsboro, OR, USA), a particle size analyzer (Zetasizer Nano ZS; Malvern, Worcestershire, UK), and western blot analysis were used to examine the morphology, size, distribution, biomarkers (HSP70 and CD9), and CagA protein level of the exosomes.

### Entrance of Exosomes Into Endothelial Cells

To determine if the exosomes entered brain endothelial cells, PKH67-labeled (green fluorescent) (Sigma-Aldrich, St. Louis, MO, USA) exosomes were added to HBMEC as described previously (30). PKH67-labeled exosomes were incubated with HBMEC for 12 h, and F-actin in the cytoskeleton was stained with Alexa Fluor 555 Phalloidin (A34055; Thermo Fisher, Waltham, MA, USA), and cell nuclei were stained with DAPI fluorescent (D1306; Thermo Fisher, Waltham, MA, USA). Cells were visualized using a confocal laser scanning microscope (Leica TCS SP8, Buffalo Grove, IL, USA).

### Endothelial Cell Proliferation, Migration, and Tube Formation Assays

Human brain microvascular endothelial cell proliferation, migration, and tube formation were assessed using a 5-ethynyl-2′-deoxyuridine (EdU) cell proliferation kit (C10337, Invitrogen, Waltham, MA, USA), a chamber transwell system (MCEP24H48, Millipore, Germany), and a Matrigel matrix (356234, Corning Life Sciences, Corning, NY, USA) according to the manufacturer's instructions (30). In brief, HBMECs (1 × 104 cells) were treated with exosomes for 24 h and stained with EdU dye and DAPI to assess cell proliferation with a fluorescence microscope. HBMECs (2 × 104 cells) were treated with exosomes for 24 h, plated in the upper transwell chamber for 8 h, fixed, and stained with crystal violet for 10 min. Migrating cells were counted in five random microscopic fields. HBMECs (2 × 104 cells) were treated with exosomes for 24 h and seeded on matrigel (70 μl) for 4 h to assess tube formation under an inverted light microscope. Total capillary tube lengths were quantified using the software Image J. Five independent fields were assessed for each well.

### Statistical Analysis

A cross-sectional study was performed to determine the association between intracranial atherosclerosis and *H. pylori* infection. Categorical variables were presented as percentages (%) and analyzed using chi-square (X^2^). Quantitative variables were presented as mean ± SE and analyzed using the one-way ANOVA. Adjusted ORs were estimated with logistic regression analysis models. A two-tailed unpaired t-test was used for the analysis of two groups of data with normal distribution and equal variance, and a two-tailed unpaired t-test with Welch correction was used to analyze two groups of data with normal distribution and unequal variance. A Mann-Whitney U test was used for comparisons between two groups of data with abnormal distributions. All data were analyzed using SPSS software (Mac Version 25.0, Chicago, IL, USA), and *p* < 0.05 was considered statistically significant.

## Results

### Factors Associated With Intracranial Atherosclerosis

A total of 15,798 subjects received screening using both TCD and a 13C-UBT. After exclusion criteria were applied, 14,084 individuals were included in the study, of whom 9,497 and 4,587 were men and women, respectively (Figure 1B). Subjects were divided into two groups, one including 4,935 patients with intracranial atherosclerosis and the other including 9,149 individuals without intracranial atherosclerosis (Table 1).

**Table 1:** Baseline characteristics for patients with and without intracranial atherosclerosis.

	**Without intracranial atherosclerosis (n = 9,149)**	**With intracranial atherosclerosis (n = 4,935)**	***p* value**
Female, n (%)	2,327 (25.4%)	2,260 (45.7%)	0.000
*H. pylori* infection, n (%)	3,220 (35.1%)	1,861 (37.7%)	0.003
Age (years)	43.45 ± 6.79	56.31 ± 8.91	0.000
Weight (kg)	68.80 ± 11.83	64.28 ± 11.05	0.000
BMI (kg/m^2^)	24.89 ± 3.30	24.73 ± 3.16	0.006
SBP (mmHg)^*^	121.81 ± 14.56	134.84 ± 19.23	0.000
DBP (mmHg)^†^	79.37 ± 11.67	82.49 ± 12.12	0.000
Hypertension, n (%)	904 (9.9%)	1,713 (34.7%)	0.000
Diabetes mellitus, n (%)	1,029 (11.2%)	1,299 (26.3%)	0.000
Smoking, n (%)	3,749 (43.6%)	1,484 (31.5%)	0.000
Alcohol, n (%)	5,039 (59.7%)	1,291 (28.8%)	0.000
Fasting blood sugar (mmol/L)	5.31 ± 1.28	5.72 ± 1.78	0.000
Glycated hemoglobin (%)	5.51 ± 0.75	5.79 ± 1.02	0.000
Total cholesterol (mmol/L)	5.11 ± 0.99	5.28 ± 1.06	0.000
Triglycerides (mmol/L)	2.11 ± 2.07	1.94 ± 1.81	0.000
HDL-cholesterol (mmol/L)^‡^	1.43 ± 0.37	1.48 ± 0.39	0.000
LDL-cholesterol (mmol/L)^§^	2.75 ± 0.84	2.94 ± 0.90	0.000

*Data were expressed as mean ± SD or n (%), where appropriate*.

* *SBP, systolic blood pressure*;

† *DBP, diastolic blood pressure*;

‡ *HDL, high-density lipoprotein*;

§ *LDL, low-density lipoprotein*.

Patients with intracranial atherosclerosis had a significantly higher rate of *H. pylori* infection, higher incidence of arterial hypertension and T2DM, higher age, and higher SBP, DBP, fasting blood sugar, glycated hemoglobin, total cholesterol (TC), high-density lipoprotein-cholesterol (HDL-C), and low-density lipoprotein-cholesterol (LDL-C) (Table 1). Patients also had a lower incidence of smoking, alcohol use, body weight, BMI, and triglycerides. There was a significantly higher proportion of women in the intracranial atherosclerosis group than the group without intracranial atherosclerosis (45.7 vs. 25.4%, *p* < 0.001; Table 1).

### Gender Differences in the Prevalence of Intracranial Atherosclerosis

Women had a significantly higher prevalence of intracranial atherosclerosis than men (49.3 vs. 28.2%, *p* < 0.001; Figure 1B). In addition, female patients with intracranial atherosclerosis had a higher rate of *H. pylori* infection, higher levels of TC, HDL-C, and LDL-C, a lower incidence of arterial hypertension and T2DM, and lower levels of weight, BMI, fasting blood sugar, SBP, DBP, and triglycerides than men with intracranial atherosclerosis (Table 2).

**Table 2:** Gender difference analysis in intracranial atherosclerosis patients.

	**Male (n = 2,675)**	**Female (n = 2,260)**	***p* value**
*H. pylori* infection, n (%)	905 (33.8%)	956 (42.3%)	0.000
Age (years)	56.50 ± 9.046	56.10 ± 8.750	0.109
Weight (kg)	69.998 ± 9.793	57.516 ± 8.395	0.000
BMI (kg/m^2^)	25.273 ± 3.067	24.099 ± 3.163	0.000
SBP (mmHg)^*^	84.69 ± 12.133	79.90 ± 11.595	0.000
DBP (mmHg)^†^	135.66 ± 18.332	133.84 ± 20.285	0.000
Hypertension, n (%)	997 (37.3%)	716 (31.7%)	0.001
Diabetes mellitus, n (%)	788 (29.5%)	511 (22.6%)	0.000
Smoking, n (%)	1,448 (59.2%)	36 (1.6%)	0.000
Alcohol, n (%)	1,113 (45.9%)	178 (8.7%)	0.000
Fasting blood sugar (mmol/L)	5.937 ± 2.076	5.470 ± 1.33	0.000
Glycated hemoglobin (%)	5.23 ± 1.96	5.22 ± 1.51	0.865
Total cholesterol (mmol/L)	5.165 ± 1.055	5.429 ± 1.039	0.000
Triglycerides (mmol/L)	2.19 ± 2.157	1.642 ± 1.221	0.000
HDL-cholesterol (mmol/L)^‡^	1.363 ± 0.342	1.624 ± 0.391	0.000
LDL-cholesterol (mmol/L)^§^	2.837 ± 0.905	3.053 ± 0.888	0.000

*Data were expressed as mean ± SD or n (%), where appropriate*.

* *SBP, systolic blood pressure*;

† *DBP, diastolic blood pressure*;

‡ *HDL, high-density lipoprotein*;

§ *LDL, low-density lipoprotein*.

### Differences in Age and Gender Among Patients With *H. pylori* Infection and Intracranial Atherosclerosis

There was a significant difference in the incidence of intracranial atherosclerosis between subjects with and without *H. pylori* infection (36.6 vs. 34.1%, *p* < 0.01; Figure 1D). *H. pylori*-positive female patients had a significantly higher incidence of intracranial atherosclerosis than *H. pylori*-negative female subjects (53.8 vs. 46.4%, *p* < 0.001). Incidence of intracranial atherosclerosis was also shown to differ by the age of female subjects with and without *H. pylori* infection (Figure 1D). However, there was no significant difference in the incidence of intracranial atherosclerosis between male subjects with and without *H. pylori* infection (Figure 1D). Further analysis showed that after age was adjusted in patients with and without intracranial atherosclerosis, *H. pylori* infection significantly increased the risk of intracranial atherosclerosis (OR = 2.261, 95% CI = 1.839–2.780, *p* < 0.001) among women <60 years old, but not among women >60 years old (Table 3, Figure 1D). *H. pylori* infection, age, BMI, DBP, arterial hypertension, TC, and glycated hemoglobin were independent factors of increased risk of intracranial atherosclerosis among women <60 years of age, while alcohol use and HDL-C were independent factors for decreased risk of intracranial atherosclerosis (Table 3).

**Table 3:** Risk factors for intracranial atherosclerosis in female patients under 60 years.

	**Odds ratio (95% CI)**	***p* value**
*H. pylori* infection, n (%)	2.261 (1.839–2.780)	0.000
Age (years)	1.262 (1.235–1.289)	0.000
Alcohol, n (%)	0.501 (0.275–0.445)	0.000
BMI (kg/m^2^)	1.175 (1.102–1.253)	0.000
SBP (mmHg)^*^	0.969 (0.954–0.984)	0.000
DBP (mmHg)^†^	1.055 (1.043–1.067)	0.000
Diabetes mellitus, n (%)	1.450 (1.177–1.786)	0.000
HDL-cholesterol (mmol/L)^‡^	0.719 (0.540–0.957)	0.024
Hypertension, n (%)	2.912 (2.056–4.125)	0.000
Total cholesterol (mmol/L)	1.198 (1.054–1.362)	0.000
Weight (kg)	0.976 (0.963–0.990)	0.001

*Data were expressed as mean ± SD or n (%), where appropriate*.

* *SBP, systolic blood pressure*;

† *DBP, diastolic blood pressure*;

‡ *HDL, high-density lipoprotein*.

### Serum Exosomes From CagA^+^ *H. pylori*-Infected Patients Inhibited Brain Endothelial Cell Proliferation, Migration, and Tube Formation

**Figure 2:**
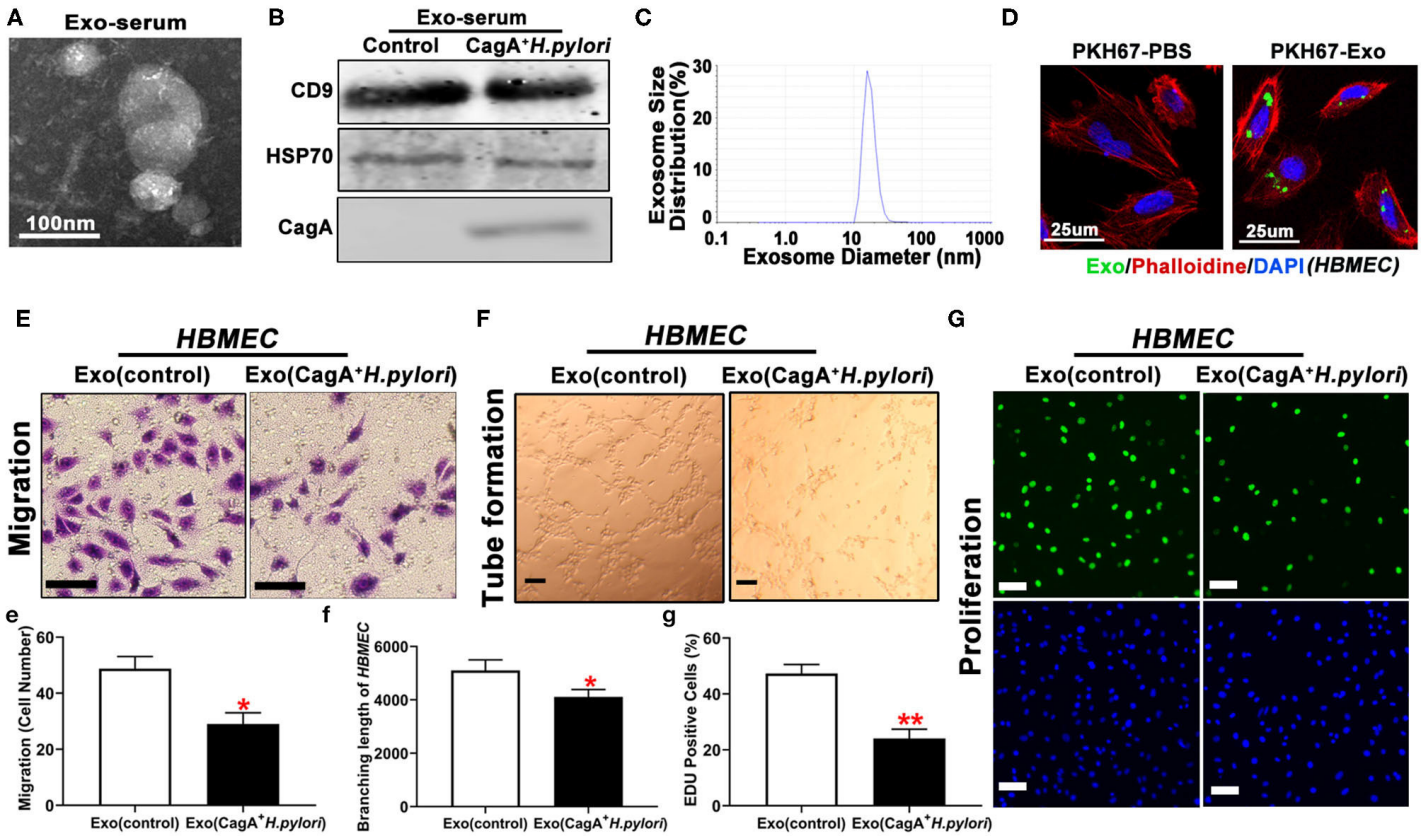
Serum exosomes from patients with CagA^+^ *H. pylori* infection inhibited endothelial function *in vitro*. Exosomes isolated from patients with CagA^+^ *H. pylori* infection exhibited typical exosome morphology **(A)**, biomarker (CD9 and HSP70) expression **(B)**, and size distribution **(C)**. Specific CagA protein presented the serum exosomes **(B)**. PKH67-labeled exosomes were tested inside the HBMEC using a confocal microscope **(D)**, indicating that the exosomes carrying CagA protein entered the HBMEC. Exposure to the CagA-containing exosomes significantly inhibited the migration **(E,e)**, tube formation **(F,f)**, and proliferation **(G,g)** of HBMEC as compared with controls (scale bar = 100 μm). Exo: exosomes. Exo(controls): serum exosomes from healthy subjects. *p < 0.05, **p < 0.01 by t-test. Data were presented as the mean ± SE. The experiment was repeated three times for every measurement. CagA, cytotoxin-associated gene A; HBMEC, human brain microvascular endothelial cells.

Endothelial dysfunction is considered the initial event in the development and progression of atherosclerosis (26). Exosomes are found in various body fluids and play an essential role in cell-to-cell communication through the transport of various bioactive constituents, such as proteins, cell-surface receptors, and microRNAs (28). A prior study showed that *H. pylori* virulence factor CagA existed in serum-derived exosomes in patients with CagA^+^ *H. pylori* infection (30). To test whether serum exosomes impacted the cell function of HBMEC, serum exosomes were isolated from human patients with CagA^+^ *H. pylori* infection or healthy control subjects by ultracentrifugation. TEM, western blotting, and particle size analysis demonstrated the presence of round-shaped vesicles measuring 10–100 nm (Figures 2A–C) in the serum exosomes. Western blotting demonstrated that serum exosomes contained CagA protein (Figure 2B). Fluorescence microscopy showed that PKH67-labeled CagA-containing exosomes were internalized by HBMEC (Figure 2D). Treatment with CagA-containing exosomes significantly inhibited HBMEC migration, tube formation, and proliferation *in vitro* (Figures 2E–G, e–g).

## Discussion

The present study demonstrated that *H. pylori* infection is common in China, with a rate of 38.8% for women and 34.8% for men (Supplementary Figure 1). After adjusting for atherosclerosis risk factors, *H. pylori* infection was shown to be an independent risk factor for intracranial atherosclerosis, especially in women ≤ 60 years. Serum exosomes from patients with CagA^+^ *H. pylori* infection significantly decreased brain endothelial cell migration, tube formation, and proliferation *in vitro*.

Atherosclerosis, a chronic syndrome leading to the thickening and hardening of artery walls, is a major cause of morbidity and mortality of stroke worldwide (8). Evidence suggests that various infectious agents, such as *H. pylori*, contribute to atherosclerosis (36) and *H. pylori* is implicated as a microbial risk factor for stroke (37, 38). However, other studies show that there is no association between *H. pylori* infection and stroke, suggesting that *H. pylori* may not be a risk factor for microcirculatory damage in the brain (18, 20, 39). Thus, the relationship between *H. pylori* infection and stroke remains controversial. This may be because of study-specific differences in sample size, population, design, and *H. pylori* prevalence. The present study showed a significant association between *H. pylori* infection and intracranial atherosclerosis, especially in women ≤ 60 years of age.

Cytotoxin-associated gene A is considered the most important *H. pylori* virulence factor (40). Many studies show that *H. pylori*, especially CagA^+^ *H. pylori* infection, contributes substantially to the risk of atherosclerosis or other cardiovascular diseases (41, 42). The current study also showed that *H. pylori* infection increased the risk of intracranial atherosclerosis. Serum exosomes from patients with CagA^+^ *H. pylori* infection significantly decreased brain endothelial function *in vitro*. However, no significant differences were seen in the risk of intracranial atherosclerosis between individuals with CagA^+^ and CagA^−^ *H. pylori* infections. This may be due to a lack of CagA-specific testing and warrants further analysis.

The underlying mechanism for the pathogenesis of *H. pylori-*associated atherosclerosis is complex and not well understood. Endothelial dysfunction is the initial event and plays a critical role in atherosclerosis (26). Evidence suggests that *H. pylori* infection could lead to increased homocysteine as a result of reduced folate and/or poor B12 absorption (43, 44). Homocysteine inhibits the secretion of nitric oxide from endothelial cells, which facilitates platelet aggregation and vasoconstriction and causes endothelial damage (45, 46). Previous studies define the relationship between *H. pylori* infection and endothelial dysfunction (30, 47–49). In the present study, serum exosomes from patients with CagA^+^ *H. pylori* infection significantly limited HBMEC function *in vitro*. These data suggested that the risk of intracranial atherosclerosis following *H. pylori* infection may be due to endothelial dysfunction. However, further studies are needed to determine if *H. pylori* infection enhances the development and/or progression of intracranial atherosclerosis via endothelial dysfunction.

Arterial hypertension, age, T2DM, and metabolic syndrome are identified as independent risk factors for intracranial atherosclerosis (8, 10, 11), and are thought to be associated with increased prevalence and severity of diseases. The present study demonstrated that patients with intracranial atherosclerosis had metabolic syndrome and had a significantly higher incidence of arterial hypertension and T2DM and higher age than those without intracranial atherosclerosis. These data are consistent with previous observations. The current study showed that patients with intracranial atherosclerosis had a lower incidence of smoking, use of alcohol, lower body weight, and BMI than those in the control group. The higher proportion of women in the intracranial atherosclerosis group may explain this difference. A multitude of investigations reports a link between *H. pylori* infection and metabolic syndrome (6, 7). In the present study, the incidence of intracranial atherosclerosis and the levels of LDL-C, TC, and SBP in patients with *H. pylori* infection were significantly higher than those without *H. pylori* infection (Supplementary Table 1). These current data showed the association between atherosclerosis and metabolic syndrome and between *H. pylori* infection and metabolic syndrome or intracranial atherosclerosis. These data are consistent with previous observations that *H. pylori* infection induces a metabolic disorder of blood lipids and a higher risk of atherosclerosis (50–52). Thus, it is speculated that *H. pylori* may induce metabolic syndrome and endothelial dysfunction, and promote the development and progression of intracranial atherosclerosis.

Gender differences exist in many cerebrovascular diseases. It is well known that dementia risk and severity are significantly higher in women than men (53–55). The incidence of subarachnoid hemorrhage (SAH) and aneurysms are higher in women than men due to hormonal differences, intrinsic wall weaknesses, and hemodynamic forces on intracranial arteries (56, 57). Women also have a higher overall lifetime risk of stroke than men (58). In the present study, women had a significantly higher prevalence of intracranial atherosclerosis than men and the intracranial atherosclerosis group had a higher proportion of women than the control group. These data were consistent with previous observations on gender differences in cerebrovascular diseases. Some studies show that *H. pylori* infection is greater in women than men, as indicated by higher bacterial load (59), other studies have shown no significant differences by sex (60, 61). In the current study, *H. pylori* infection rate was significantly higher in women than men. It is speculated that *H. pylori* infection acting as an independent risk factor for intracranial atherosclerosis in women may due to higher *H. pylori* infection rate and the increased *H. pylori* load in women.

Evidence also indicates that neurological diseases improve through the eradication of *H. pylori* infection (62–67). Patients with Parkinson's disease for whom *H. pylori* has been eradicated have reduced motor fluctuation (62), improved L-dopa absorption (63), and slower disease progression (64). Some studies observed an improvement in cognitive and functional status (65) and a decreased disease progression (66) in Alzheimer's disease patients after the eradication of *H. pylori*. Another study showed that 6 months after eradication of *H. pylori* in ischemic stroke patients, plasma levels of TC, LDL-C, fibrinogen, and IL-8 were significantly lower than those seen in *H. pylori*-infected stroke patients and controls (67). Future studies are needed to investigate a potential association between *H. pylori-*eradication and intracranial atherosclerosis.

There were a few limitations in this study. The study cohort was enrolled from one center with individuals of the same ethnicity. In addition, findings could not elucidate the long-term influence of *H. pylori* infection on intracranial atherosclerosis because of a lack of follow-up data. Future prospective studies are needed to determine if significant gender differences are observed over time.

## Conclusion

In summary, *H. pylori* infection acted as an independent risk factor for intracranial atherosclerosis in women ≤ 60 years of age but not among older women or men.

## Data Availability Statement

The raw data supporting the conclusions of this article will be made available by the authors, without undue reservation.

## Ethics Statement

The studies involving human participants were reviewed and approved by the Clinical Research Ethics Committee of the Third Xiangya Hospital of Central South University, Changsha, China (Approval Number of Ethics Committee: 2019-S241). The patients/participants provided their written informed consent to participate in this study.

## Author Contributions

XX and ZC were in charge of the entire project and revised the draft of the manuscript. YG collected the data and wrote the manuscript. CX and LZ did the statistics. All authors contributed to the article and approved the submitted version.

## Funding

This work was supported by grants from Fundamental Research Funds for the Central Universities of Central South University (Grant No. 2019zzts352) and the National Innovative Training Program of China (Grant No. 201310533059).

## Conflict of Interest

The authors declare that the research was conducted in the absence of any commercial or financial relationships that could be construed as apotential conflict of interest.

## Publisher's Note

All claims expressed in this article are solely those of the authors and do not necessarily represent those of their affiliated organizations, or those of the publisher, the editors and the reviewers. Any product that may be evaluated in this article, or claim that may be made by its manufacturer, is not guaranteed or endorsed by the publisher.

## Supplementary Material

Supplementary Table 1 Baseline characteristics for subjects with and without *H. pylori* Infection.

Supplementary Figure 1 Gender difference of *H. pylori* infection.
