# Supplementary material for: Helicobacter pylori Infection Acts as an Independent Risk Factor for Intracranial Atherosclerosis in Women Less Than 60 Years Old
Source: Front Cardiovasc Med. 2022 Jan 11;8:819315. doi: 10.3389/fcvm.2021.819315 (PMC8787118; doi:10.3389/fcvm.2021.819315)
Supplement: Supplementary Table 1 — Baseline characteristics for subjects with and without H. pylori Infection. [file Data_Sheet_1.docx]

Supplementary Material

# Supplementary Table and Figure

## Supplementary Table 1. Baseline characteristics for subjects with and without *H. pylori* infection.

|  | Without *H.pylori* Infection  (n=9003) | With *H.pylori* Infection  (n=5081) | *p* Value |
| --- | --- | --- | --- |
| Intracranial atherosclerosis, n(%) | 3074(34.1%) | 1861(36.6%) | 0.003 |
| Female, n(%) | 2809(31.2%) | 1778(35%) | 0.000 |
| Age(years) | 47.94±10.066 | 48±9.225 | 0.742 |
| Weight(Kg) | 67.243±11.708 | 67.183±11.868 | 0.770 |
| BMI(Kg/m^2^) | 24.813±3.283 | 24.882±3.216 | 0.229 |
| SBP(mmHg)* | 126.09±17.28 | 126.87±17.87 | 0.012 |
| DBP(mmHg)† | 80.34±11.82 | 80.70±112.11 | 0.084 |
| Hypertension, n(%) | 1668 (18.4%) | 948(18.7%) | 0.840 |
| Diabetes mellitus, n(%) | 1387(15.4%) | 940(18.5%) | 0.000 |
| Smoking, n(%) | 3428(37.9%) | 1804(35.5%) | 0.001 |
| Alcohol, n(%) | 3936(43.5%) | 2393(47.1%) | 0.000 |
| Fasting blood sugar (mmol/L) | 5.450±1.461 | 5.466±1.554 | 0.545 |
| Glycated hemoglobin(%) | 5.615±0.840 | 5.589±0.882 | 0.000 |
| Total cholesterol (mmol/L) | 5.157±1.003 | 5.199±1.046 | 0.019 |
| Triglycerides (mmol/L) | 2.074±1.988 | 2.013±1.981 | 0.080 |
| HDL-cholesterol (mmol/L)‡ | 1.450±0.375 | 1.440±0.376 | 0.155 |
| LDL-cholesterol (mmol/L)§ | 2.791±0.862 | 2.861±0.883 | 0.000 |

Data were expressed as mean±SD or n (%), where appropriate. *SBP: systolic blood pressure; †DBP: diastolic blood pressure; ‡HDL: high-density lipoprotein; §LDL: Low-density lipoprotein.

## Supplementary Figure


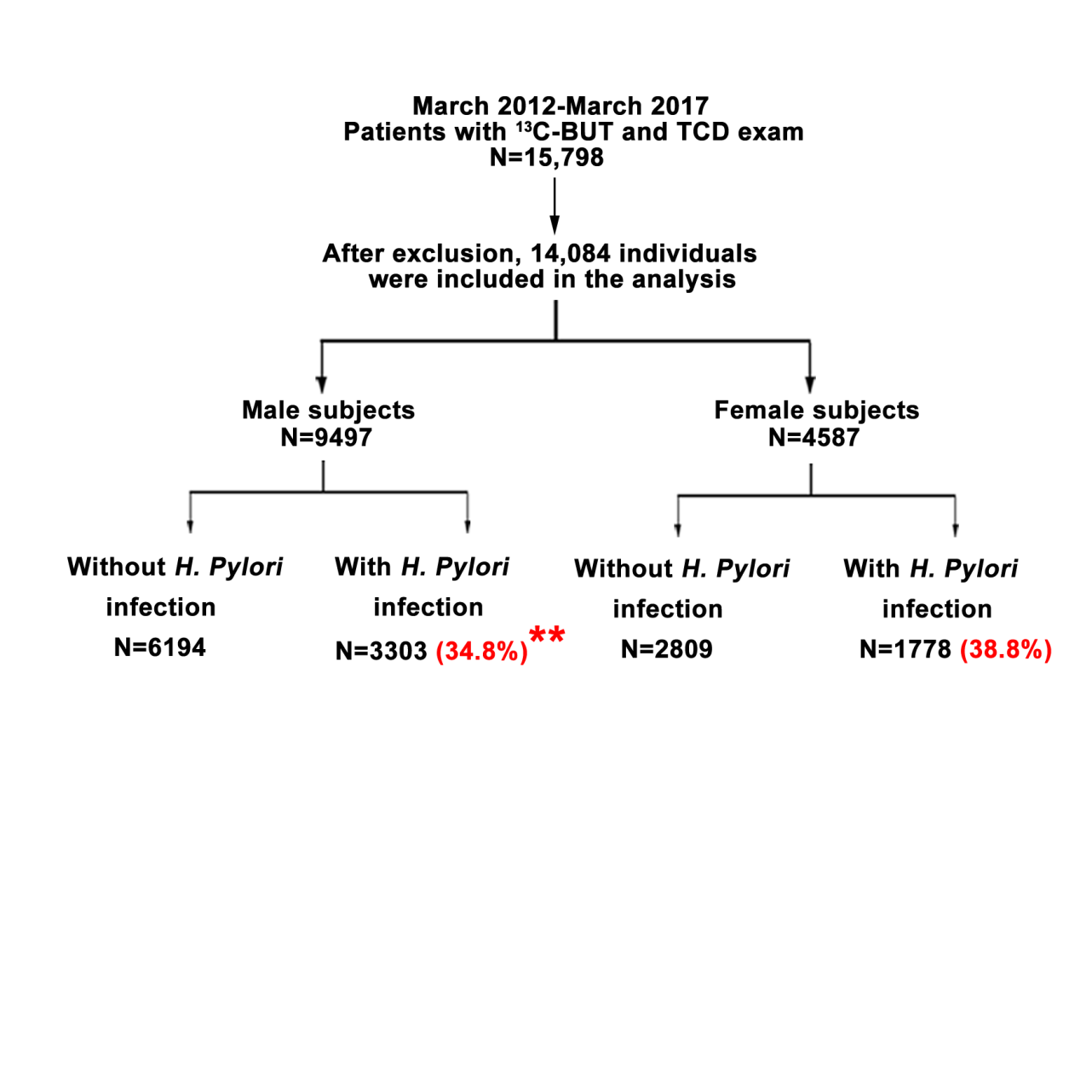


**Supplementary Figure 1. Gender difference of *H. pylori* infection.** The rate of *H. pylori* infection is significantly higher in female subjects than males (38.8% vs. 34.8%, ***p*<0.01).
